# Binding of Histone H1 to DNA Is Differentially Modulated by Redox State of HMGB1

**DOI:** 10.1371/journal.pone.0089070

**Published:** 2014-02-13

**Authors:** Eva Polanská, Šárka Pospíšilová, Michal Štros

**Affiliations:** 1 Laboratory of Analysis of Chromosomal Proteins, Institute of Biophysics, Academy of Science of the Czech Republic, Brno, Czech Republic; 2 Central European Institute of Technology (CEITEC) – Center of Molecular Medicine, Masaryk University, Brno, Czech Republic; National Cancer Institute, United States of America

## Abstract

HMGB1 is an architectural protein in chromatin, acting also as a signaling molecule outside the cell. Recent reports from several laboratories provided evidence that a number of both the intracellular and extracellular functions of HMGB1 may depend on redox-sensitive cysteine residues of the protein. In this study we demonstrate that redox state of HMGB1 can significantly modulate the ability of the protein to bind and bend DNA, as well as to promote DNA end-joining. We also report a high affinity binding of histone H1 to hemicatenated DNA loops and DNA minicircles. Finally, we show that reduced HMGB1 can readily displace histone H1 from DNA, while oxidized HMGB1 has limited capacity for H1 displacement. Our results suggested a novel mechanism for the HMGB1-mediated modulation of histone H1 binding to DNA. Possible biological consequences of linker histones H1 replacement by HMGB1 for the functioning of chromatin are discussed.

## Introduction

Chromatin-associated protein HMGB1 has been implicated in DNA replication, recombination, repair and transcription, as well as in cell signaling, promotion of tumor growth and metastasis (reviewed in [1]). HMGB1 can also act as an extracellular damage associated molecular pattern molecule (DAMP), regulating cell death and survival [2], [3]. The importance of HMGB1 protein for life was revealed from knockout experiments demonstrating that inactivation of the *HMGB1* gene in mice was lethal [4]. Recent reports from several laboratories provided evidence that both intracellular and extracellular functions of HMGB1 may depend on redox-sensitive cysteine residues of the protein (reviewed in [2]). Redox state of HMGB1 is also critical for the nucleocytoplasmic shuttling of the protein [5]. All-thiol (reduced) HMGB1 acts as a chemoattractant, but a disuphide bond within (oxidized) HMGB1 can turn it into a proinflammatory cytokine ([6] and refs. therein). It has also been shown that redox state (oxidation or modification of cysteine residues) of HMGB1 could impair the binding affinity of the protein to linear cisplatin-modified DNA or superhelical DNA [7]–[10].

The histone H1 family represents an important class of structural and architectural proteins that are responsible for maintaining and stabilizing higher-order chromatin structure. H1 histones are also responsible for gene-specific regulation of transcription and other DNA-dependent processes (reviewed in [11]). Eleven different H1 subtypes have been identified in mammals, seven of them are somatic: H1.1–H1.5, H1^o^ and H1x [11]. Although knock-out of one or two of the somatic *H1* genes is dispensable for normal mouse development (including histone H1^o^, [12]), knock-out of three *H1* genes (*H1.2, H1.3 and H1.4*) is lethal [13].

The non-sequence specific proteins H1 and HMGB1 share binding preference to alternative DNA structures, such as bent, kinked and unwound DNA structures (e.g., four-way junctions, supercoiled DNA and cisPt-modified DNA, reviewed in [1]). In addition, both proteins occupy similar (if not identical) binding sites within the linker DNA in chromatin (reviewed in [1]). A number of data indicated that linker H1 histones could be excluded or displaced from DNA or chromatin by HMGB-type proteins [14]–[17]. These and other data (refs. in [18]) suggested that the presence of H1 and HMGB1 in chromatin might be mutually exclusive. The displacement of histones H1 from their binding site could have important biological consequences including local destabilization of chromatin, recruitment of other proteins, and transcriptional activation ([18] and refs. therein). While post-transtranslational modifications can fine-tune binding affinity of H1 and HMGB1 for DNA and chromatin (reviewed in [1]), the precise mechanism how HMGB1 could (reversibly) modulate histone H1 binding to DNA is far from clear.

In this study we demonstrate that redox state of HMGB1 can significantly modulate the ability of the protein to bind and bend DNA, as well as to promote DNA end-joining. We also report a high affinity binding of histone H1 to small DNA circles and hemicatenated DNA loops. Finally, we show that reduced HMGB1 can readily displace histone H1 from DNA, while oxidized HMGB1 has limited capacity for H1 displacement. Our results suggests a novel mechanism for HMGB1-mediated modulation of histone H1 binding to DNA.

## Results

### Redox State-dependent Modulation of DNA Bending and DNA End-joining by HMGB1

Reports from several laboratories provided evidence that both intracellular and extracellular functions of HMGB1 may depend on redox-sensitive cysteine residues of the protein (Cys23, Cys45 and Cys106, reviewed in [2]). It has also been reported that oxidation or modification of cysteine residues of HMGB1 could impair the binding affinity of the protein to linear or bent DNA such as cisplatin-modified or superhelical DNA [7]–[10].

#### Oxidization of HMGB1

**Figure 1:**
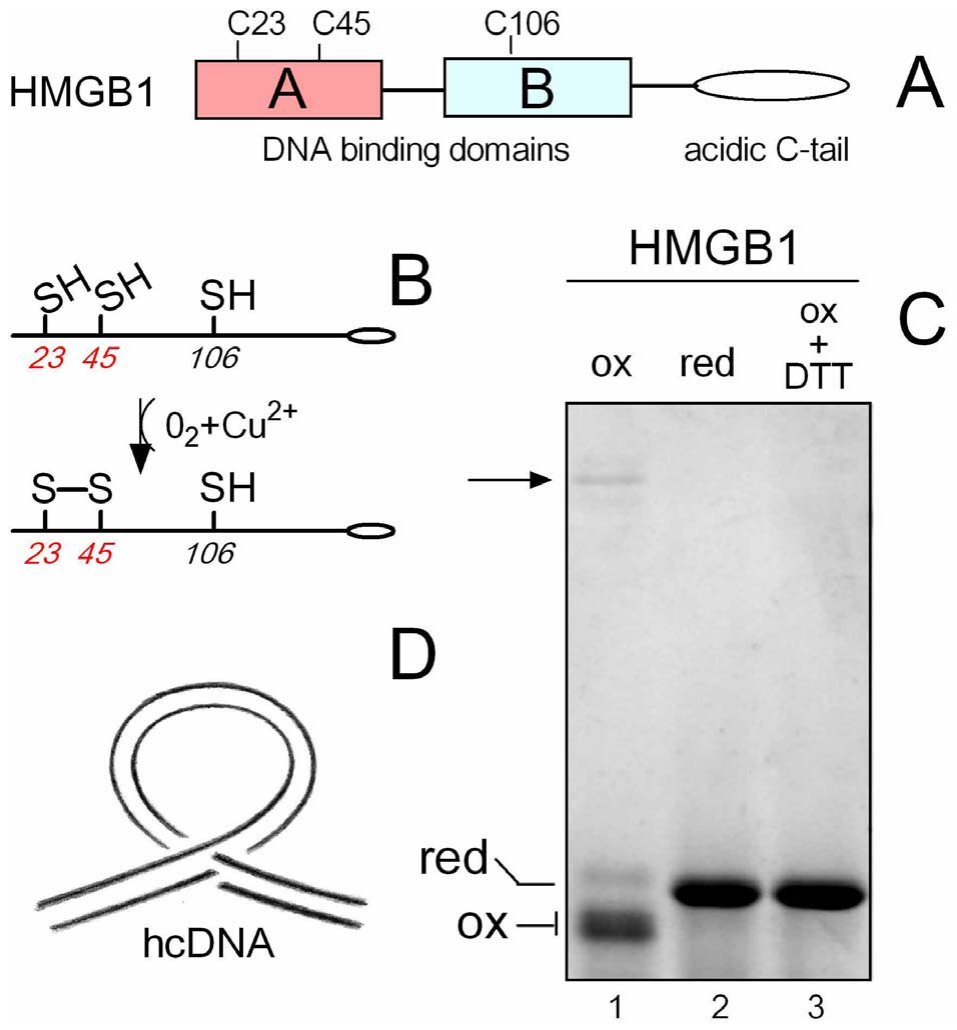
Domain structure of HMGB1, cysteine residues of HMGB1 and oxidation, hcDNA. (**A**) Domain structure of HMGB1 with two DNA binding domains, and polyanionic C-tail. (**B**) Schematic representation of cysteine residues of HMGB1 and formation of a disulphide bridge between Cys23 and Cys45. (**C**) Mild oxidation of HMGB1 in the presence of Cu^2+^ results in increased mobility in PAGE due to formation of an intramolecular disulphide bond by opposing Cys23 and Cys45, in agreement with MALDI-TOF mass spectrometry. Equal amounts (4 µg) of oxidized or reduced HMGB1 samples were loaded on the SDS-15%-polyacrylamide gel. Notice that oxidization of HMGB1 compromised Coomassie blue-staining of the protein in the gel. Arrow indicates electrophoretic mobility of HMGB1 dimer (formed due to an intermolecular cross-link of two HMGB1 molecules via the disulphide bridge). (**D**) Schematic drawing of hemicatenated DNA loops (hcDNA). hcDNA was created from a sequence containing a tract of poly(CA)·poly(TG) that can form a loop maintained at its base by hemicatenane, i.e. the junction of two DNA duplexes in which one of the strands of one duplex passes between the two strands of the other duplex [26], [42]. The drawing was kindly provided by François Strauss (National Museum of Natural History, Paris, France).

HMGB1 was subjected to mild oxidization by dialysis against 5 µM Cu^2+^ and re-dialysis against buffer without Cu^2+^ as previously reported [5], [9]. As shown in Figure 1C, electrophoretic mobility of the oxidized HMGB1 was enhanced relative to the reduced form of the protein [Figure 1C, notice that oxidization of HMGB1 brought about lower staining of the protein by Coomassie blue in the polyacrylamide gel (lane 1). However, treatment of the oxidized HMGB1 with DTT (lane 3) resulted in identical HMGB1 staining to that previously observed with reduced protein (lane 2), demonstrating equal loading of the oxidized and reduced HMGB1 proteins on the gel]. The appearance of the faster moving band upon HMGB1 oxidation was most likely due to formation of an intramolecular disulphide bond by opposing Cys23 and Cys45 (Figure 1B). The presence of a disulphide bridge between Cys23 and Cys45 was confirmed by MALDI-TOF mass spectrometry of trypsin digested HMGB1 samples. No other amino acids of HMGB1 were affected by mild oxidization, with the exception of oxidization of a small fraction of Cyst106 to sulphenic acid. Partial modification of C106 to sulphenic acid could not significantly influence the results described in this paper as all binding experiments performed with mildly oxidized wt HMGB1 or HMGB1(C106S) mutant were indistinguishable (not shown).

**Figure 2:**
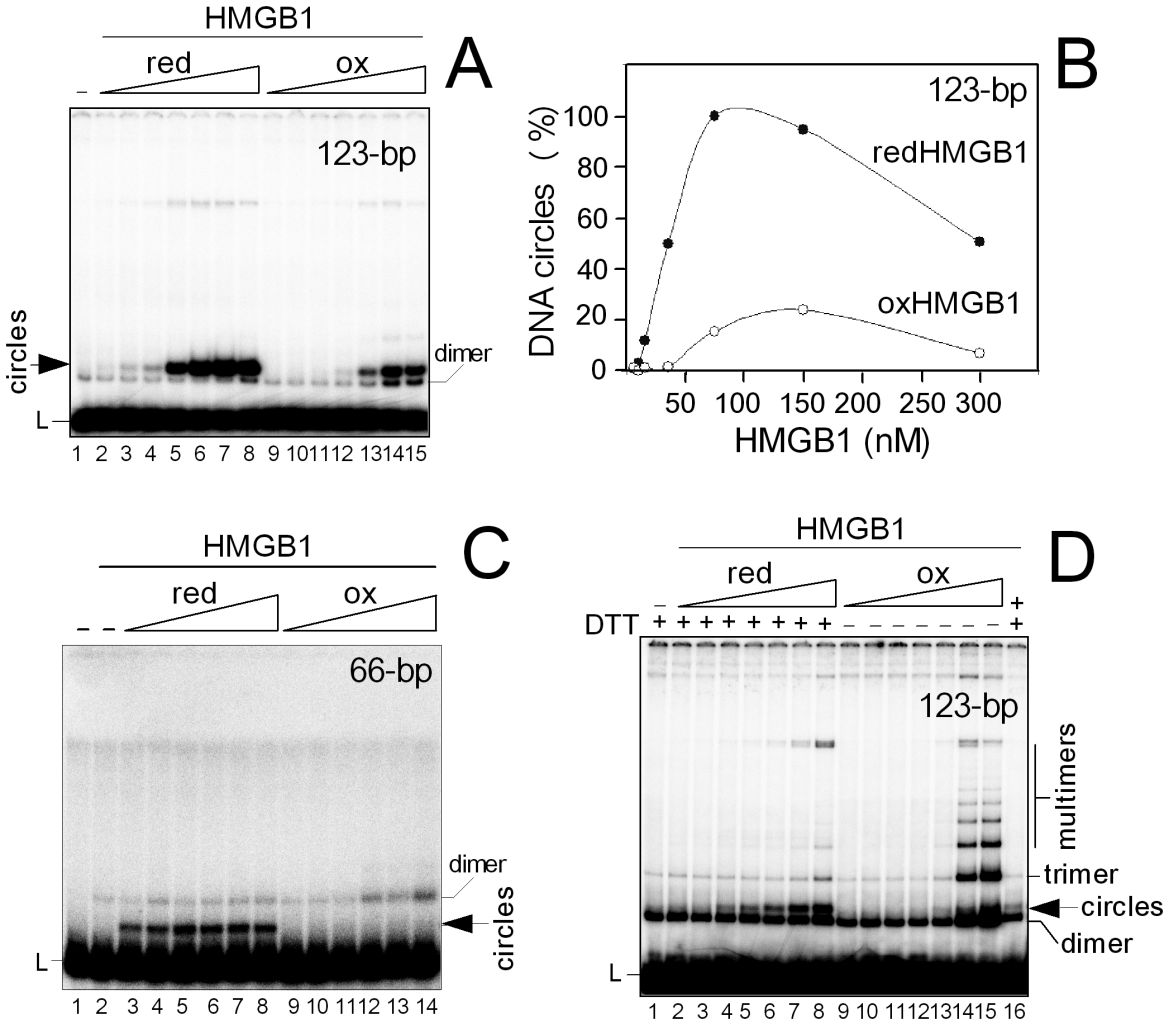
Redox state-dependent modulation of DNA bending and end-joining by HMGB1. (**A**) DNA bending by HMGB1. ^32^P-labeled 123-bp DNA duplex (∼0.2 nM) was pre-incubated with different amounts of reduced or oxidized HMGB1 (6, 10, 15, 35, 75, 150 and 300 nM; lanes 2–8), followed by ligation with T4 DNA ligase as detailed under “*Materials and Methods*”. (**B**) Percentage of DNA minicircles from ligase-mediated circularization assay in the presence of reduced or oxidized HMGB1. 100% denotes production of DNA minicircles at 75 nM reduced HMGB1 protein. Presented data are based on four independent experiments. redHMGB1, reduced HMGB1; oxHMGB1, oxidized HMGB1. (**C**) DNA bending by HMGB1. ^32^P-labeled 66-bp DNA duplex (∼1 nM) was pre-incubated with different amounts of reduced or oxidized HMGB1 (50, 100, 150, 200, 250 and 400 nM; lanes 3–8), followed by ligation with T4 DNA ligase as detailed under “Materials and Methods”. Lane 1, no ligase added. (**D**) DNA end-joining by HMGB1. The ^32^P-labeled 123-bp DNA fragment (∼2 nM) was pre-incubated with different amounts of reduced or oxidized HMGB1 (10, 20, 40, 60, 100, 150 and 200 nM; lanes 2–8), followed by ligation with T4 DNA ligase as in panels (**A**) and (**B**). Lane 16 in panel (**D**) corresponds to the reaction in lane 14 in which the oxidized HMGB1 was pre-treated with 10 mM DTT before addition of ligase. L, linear 123-bp DNA duplex.

We have several piece of evidence that mild oxidization of HMGB1 by Cu^2+^/O_2_ was fully reversible. First, treatment of the oxidized HMGB1 protein with 10 mM DTT could reverse the electrophoretic mobility to that of the reduced protein (Figure 1, lanes 1 and 3). Second, binding and bending properties of the DTT-treated oxidized HMGB1 were similar to that of reduced HMGB1 (Figure 2). Finally, MALDI-TOF analysis revealed the absence of the disulphide bridge between Cys25 and Cys45 of the DTT-treated oxidized HMGB1 (not shown). Thus, all-thiol- and disulfide-bond-containing HMGB1 proteins (i.e., reduced and oxidized) are readily interconverted in the presence of electron donors (DTT) or acceptors (oxygen in the presence of Cu^2+^).

#### Redox state-dependent DNA bending by HMGB1

DNA bending/looping by HMGB1 provides a possible mechanism by which the protein promotes activity of various gene promoters by enhancement of binding of transcription factors and/or bringing distant regulatory sequences into close proximity (reviewed in [1]). Here, we have investigated whether DNA bending by HMGB1 could be modulated by the redox state of the protein. We have used ligase-mediated circularization assay to measure the efficiency with which T4 DNA ligase forms circles from short DNA fragments by HMGB1 protein [19]. In the absence of DNA curvature, the stiffness of a short DNA fragment (<150-bp) prevents intramolecular alignment of its ends so that circles are detected only in a presence of protein that bends DNA [19]. As oxidation of HMGB1 can significantly impair DNA binding properties of the protein ([7]–[10], and this paper), we have studied whether the ability of HMGB1 to bend DNA is modulated by the redox state of the protein. At low concentration of the 123-bp DNA duplex (∼0.2 nM), production of the ligase-mediated DNA minicircles peaked at 75–150 nM concentration of reduced HMGB1. Higher amounts of reduced HMGB1 resulted in decrease in formation of DNA minicircles (Figure 2A/B). On the other hand, percentage of DNA minicircles at any concentration of oxidized HMGB1 was not higher than ∼20% of that obtained with the reduced form of the protein (Figure 2A/B). Similarly to experiments with reduced HMGB1, higher amounts of oxidized HMGB1 protein had inhibitory effect on formation of minicircles (Figure 2A/B). This decrease was probably due to formation of large HMGB-DNA complexes, limiting the access of ligase to DNA (see also [20] [21]). Redox state-dependent modulation of the ability of HMGB1 to bend DNA was even more prominent in experiments employing the 66-bp DNA demonstrating the inability of the oxidized HMGB1 (unlike its reduced form) to promote DNA circularization (Figure 2C).

#### Redox state-dependent DNA end-joining by HMGB1

DNA end-joining represents one of the mechanisms for repair of double-strand breaks in eukaryotic cells via NHEJ pathway (reviewed in [22]). Previously we have discovered the ability of HMGB1 to promote DNA end-joining by T4 DNA ligase and human DNA ligase I [19], [20], and similar finding was reported with human DNA ligase IV [23]. Here, we have investigated whether the ligase-mediated formation of linear DNA multimers (i.e, DNA end-joining) could be also modulated by the redox state of HMGB1. Experiments were performed essentially as in Figure 2A but at a higher concentration of the 123-bp DNA duplex to promote formation of linear multimers in the course of DNA ligation. Under the conditions of our experiment (≥2 nM DNA), oxidized HMGB1 could enhance ∼4–6-fold production of linear multimers, relative to the effect of the reduced HMGB1 protein (Figure 2D). Treatment of the oxidized HMGB1 with 10 mM DTT inhibited the ability of the protein to promote ligase-mediated DNA end-joining (Figure 2D, lanes 14 and 16), demonstrating that the effect of oxidization on the ability of HMGB1 to promote DNA end-joining was completely reversible. We conclude that oxidized HMGB1 significantly promote ligase-mediated DNA end-joining and much less DNA bending whereas reduced HMGB1 can act in the opposite way. Thus, the ability of HMGB1 to bend DNA and promote DNA end-joining depends on the redox state of HMGB1.

### The Impact of Redox State of HMGB1 on Binding of the Protein to Small DNA Circles and Hemicatenated DNA Loops

#### HMGB1 binding to small DNA circles

**Figure 3:**
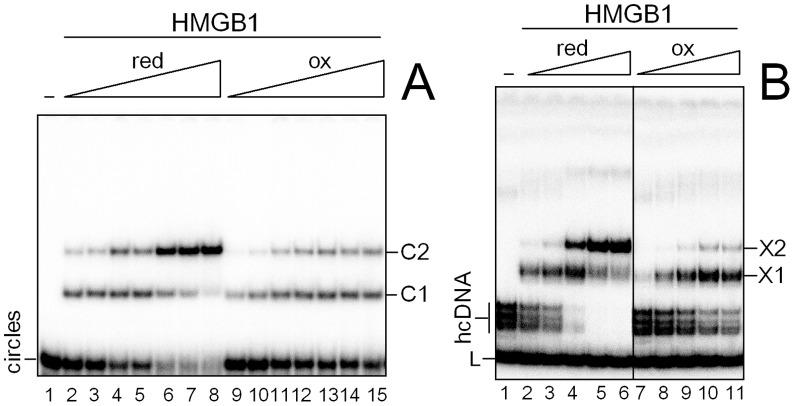
Redox state-dependent interaction of HMGB1 with DNA. (**A**) Interaction of HMGB1 with small DNA circles. Increasing amounts of reduced or oxidized HMGB1 (typically 25–800 pM; lanes 2–8 and 9–15, respectively) were added to ^32^P-labeled DNA minicircles (∼30 pM). C1 and C2 indicate specific complexes of HMGB1 with DNA minicircles. (**B**) Interaction of HMGB1 with hcDNA. Increasing amounts of reduced or oxidized HMGB1 (typically 5 to 300 pM; lanes 2–6 and 7–11, respectively) were added to ^32^P-labeled hcDNA (1.5 pM). X1 and X2 indicate the specific HMGB1-hcDNA complexes. Protein-DNA complexes were resolved on 8% (panel **A**) or 6% (panel **B**) polyacrylamide gels in 0.5x TBE and visualized by autoradiography. Representative pictures of at least four experiments are shown. *K*_d_ values for redHMGB1 and oxHMGB1 proteins were estimated as detailed in the “*Materials and Methods*”. redHMGB1, reduced HMGB1; oxHMGB1, oxidized HMGB1.

Previous reports demonstrated that small DNA minicircles of the length below the persistence length (<150-bp) were high-affinity binding substrates for a number of HMG-box proteins, including HMGB1 ([24], reviewed in [1]). Here, we have studied the impact of the redox state of HMGB1 on the interaction of the protein with DNA minicirles of 66-bp. As shown in Figure 3A, HMGB1 could bind to small DNA circles forming two well-defined complexes, C1 and C2, and the binding was clearly cooperative (in agreement with previous report [24]). *K*_d_ for HMGB1 binding to small DNA circles was estimated from EMSA by using fixed concentration of the labeled DNA and varying amounts of the proteins at the point in the titration where half of the input DNA had been complexed with protein (i.e. protein concentration at which 50% of the DNA was shifted), and calculated using the formula [P] = *K*_d_+[D]/2 where [P] and [D] are the total protein and DNA concentrations, respectively (*K_d_*, dissociation constant). The affinity of oxidized HMGB1 for small DNA circles was only ∼2-fold decreased (*K*_d_ *∼* 0.25 nM) relative to the affinity of the reduced protein (*K*_d_ *<*0.1 nM). DNA minicircles represent pre-bent and highly constrained DNA structure, with no need for further distortion as a prerequisite (or consequence) of HMGB1 binding. Therefore, the (redox state-dependent) ability of HMGB1 to bend DNA (Figure 2) is most likely dispensable in assessment of the binding potential of the protein to the small DNA circles.

#### HMGB1 binding to hemicatenated DNA loops

To find out the impact of the redox-state of HMGB1 on binding of the protein to other (bent) DNA structure, we have studied binding of reduced and oxidized HMGB1 to hemicatenated DNA loops (hcDNA). hcDNA was originally discovered by Gaillard and Strauss [25] by re-association of the strands of a DNA fragment containing a track of repetitive poly(CA).poly(TG) sequence from CA-microsatellite. The repetitive sequence is arranged in hcDNA in a DNA loop at the base of which the two DNA duplexes cross, with one of the strands of one duplex passing between the strands of the other duplex forming a DNA hemicatenane (Figure 1D). hcDNA has been previously shown to be specifically recognized and bound by HMGB1 [26]–[28]. As shown in Figure 3, both reduced HMGB1 and oxidized HMGB1 could bind to hcDNA by formation of two distinct bands, X1 and X2. Titration of hcDNA by HMGB1 revealed that the affinity of oxidized HMGB1 for hcDNA was up to 10-fold decreased than that of the reduced protein Figure 3B). Complete restoration of the binding properties of oxidized HMGB1 to hcDNA was demonstrated upon treatment of the protein with 10 mM DTT, resulting in similar binding affinity for hcDNA, previously observed with reduced HMGB1 (*K*_d_ <10 pM, data not shown). Thus, our data provided evidence that changes in DNA-binding properties upon oxidization of HMGB1 were completely reversible.

### High-affinity Binding of Histone H1 to Hemicatenated DNA Loops and Small DNA Circles

Previous reports demonstrated that both chromatin-associated proteins HMGB1 and histone H1 exhibit preferential binding to bent, distorted and unwound DNA structures (e.g., four-way junctions, supercoiled DNA and cisPt-modified DNA, reviewed in [1], [29]. Here, we have studied whether histone H1 can also bind to hemicatenated DNA loops (hcDNA) or small DNA circles.

#### Binding of H1 to hemicatenated DNA loops

**Figure 4:**
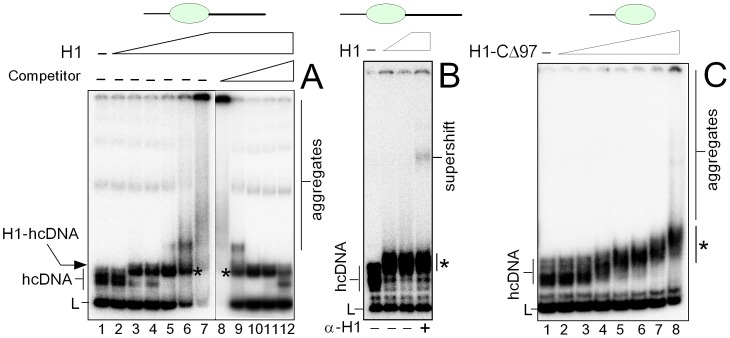
High affinity binding of human histone H1 to hemicatenated DNA loops. (**A**) Binding of H1 to hcDNA. ^32^P-labeled hcDNA **(∼**15 pM) was titrated with increasing amounts of histone H1 (3, 6, 9, 12, 18, and 36 nM, *lanes* 2–7) in the absence of competitor DNA. The H1-hcDNA complex at 36 nM H1 in the presence of increasing amounts of competitor linear DNA (10-, 10^2^-, 10^3^-, 10^4^-, and 5×10^4^-fold mass excess of unlabeled linear DNA over ^32^P-labeled hcDNA; *lanes* 8–12, *left to right*). (**B**) Detection of H1 binding to hcDNA by specific anti-H1 antibody. Gel loading (*left to right*): hcDNA, hcDNA with 2.5 or 5 nM H1. H1-hcDNA complex (at 5 nM H1) with anti-H1 antibody (indicated by +). Binding experiments were performed at 10^3^-fold excess of unlabeled competitor DNA. (**C**) Binding of H1-CΔ97 to hcDNA. ^32^P-labeled hcDNA **(∼**15 pM) was titrated with increasing amounts of histone H1 lacking the C-terminal domain (peptide H1-CΔ97) (8, 20, 40, 60, 80, 120 and 240 nM, lanes 2–8) in the absence of competitor linear DNA. The H1-DNA complexes were resolved on 8% or 6% polyacrylamide gels in 0.5x TBE and visualized by autoradiography as detailed in the “*Materials and Methods*”. Asterisks indicate the H1-hcDNA complexes.

To find out whether histone H1 could bind to hemicatenated DNA loops (hcDNA), increasing amounts of H1 were added to this DNA and the complexes were resolved by non-denaturing polycrylamide gel electrophoresis. As shown in Figure 4A (lanes 3–6), a complex with lower mobility than that of the protein-free hcDNA was observed upon titration of hcDNA with histone H1. Binding of H1 to hcDNA was verified by super-shift of the H1-hcDNA complex with specific anti-H1 antibody (Figure 4B, lower accessibility of the H1 epitope in the complex with DNA may explain that only a very small fraction of the H1-hcDNA complex was supershifted with anti-H1 antibody). We have estimated that the *K*_d_ value for H1 binding to hcDNA was ∼6 nM. Higher amounts of H1 (≥20 nM) lead to aggregation of the H1-DNA complex (Figure 4A, lanes 6–7). The aggregation was suppressed in the presence of ≥100-fold excess of unlabeled competitor DNA, and the H1-hcDNA complexes were detected up to ∼10^4^-fold excess of competitor DNA (Figure 4A, lanes 8–12).

Histone H1 has a three-domain structure: a short N-terminal domain (20–35 amino acids), a conserved globular domain (∼80 amino acids) and an extended C-terminal domain (∼100 amino acids). While the globular domain directs structure-specific recognition and binding to the nucleosome, the extremely basic C-terminal domain (CTD) is responsible for high affinity binding of H1 to chromatin *in vivo* ([30] and refs. therein). The possible importance of the extremely basic C-terminal domain (CTD) of histone H1 for specific binding of the protein to hcDNA was assessed with H1 lacking the C-terminal amino acid residues 97–193 (designated as to H1-CΔ97, see ref. [31]). As shown in Figure 4C, a gradual retardation of the (H1-CΔ97)-hcDNA complex was observed. We have estimated that the affinity of the H1 peptide for hcDNA was more than ∼6-fold lower (*K_D_* ∼35 nM) than that of the full-length H1. Specificity of H1-CΔ97 binding to hcDNA was further challenged with unlabeled competitor DNA. The (H1-CΔ97)-DNA complex proved to be stable only up to ∼1000-fold mass excess of competitor DNA (not shown). These data indicated that the CTD of histone H1 was required for both specific binding and high-affinity of the protein to hemicatenated DNA loops.

#### Binding of H1 to DNA minicircles

**Figure 5:**
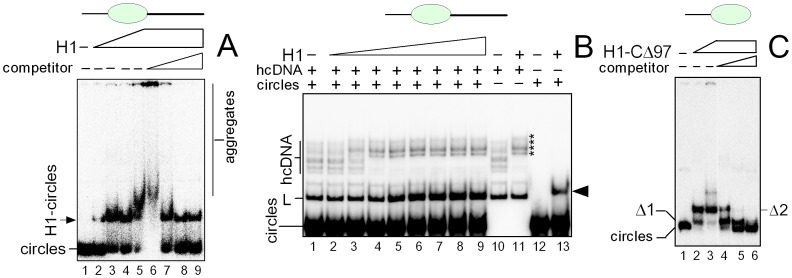
Binding of histone H1 to DNA minicircles. (**A**) Titration of DNA minicircles with histone H1. ^32^P-labeled DNA minicircles of 66-bp (∼30 pM) were titrated with histone H1 (2, 4, 8 and 15 nM, lanes 2–5) in the absence of competitor DNA. The H1-minicircles complex (prepared at 15 nM H1, lane 5) was also titrated with increasing amount competitor λ-DNA (10, 10^2^, 10^3^ and 10^4^-fold mass excess of unlabeled competitor DNA over ^32^P-labeled minicircles; lanes 6–9, *left to right*). L, linear DNA of 66-bp. (**B**) Competition of DNA minicircles for histone H1 binding to hcDNA. An equimolar mixture of ^32^P-labeled DNA minicircles (66-bp) and hcDNA (∼30 pM) was titrated with histone H1 (2, 6, 9, 12, 18, 30, 50, 80 nM, lanes 2–9). ^32^P-labeled hcDNA without (lane 10) or with (lane 11) 15 nM H1 (the H1-hcDNA complexes are indicated by asterisks). ^32^P-labeled DNA minicircles without (lane 12) or with (lane 13) 15 nM H1. Arrowhead indicates position of the H1-DNA minicircles complex. (**C**) Binding of H1-CΔ197 to DNA minicircles. ^32^P-labeled DNA minicircles **(∼**30 pM) were titrated with increasing amounts of histone H1 lacking the C-terminal domain (peptide H1-CΔ97) (10 and 20 nM, *lanes* 2 and 3) in the absence of competitor DNA. Complex (H1-CΔ97)-DNA minicircles (prepared at 20 nM H1 peptide, lane 3) was also titrated with increasing amounts of competitor λ-DNA (10^3^, 10^4^ and 10^5^-fold mass excess of unlabeled competitor DNA over ^32^P-labeled DNA minicircles, lanes 4–6, *left to right*). Δ1 and Δ2 indicate the (H1-CΔ97)-DNA minicircles complexes. H1-DNA complexes were resolved on 8% or 6% polyacrylamide gels in 0.5×TBE and visualized by autoradiography as detailed in the “*Materials and Methods*”.

To find out whether histone H1 has a potential to bind to the DNA loop within hcDNA, DNA minicircles of 66-bp (a size similar to that of the DNA loop within hcDNA) were titrated with increasing amounts of H1. As shown in Figure 5A, addition of increasing amounts of human histone H1 to ^32^P-labeled DNA minicircles of 66-bp (in the absence of unlabeled competitor DNA) resulted in the appearance of a single complex of reduced mobility, the intensity of which increased up to ∼10 nM concentration of the protein. We have estimated that the *K*_d_ value for H1 binding to DNA minicircles was ∼5 nM. Higher amounts of H1 (>10 nM) lead to aggregation of the H1-minicircles complex if no competitor DNA was present (Figure 5A). Similarly to H1 binding to hcDNA, the aggregation of the H1-DNA minicircles complex was fully suppressed in the presence of ≥100-fold excess of competitor DNA, and the complex was detected up to ∼10^4^-fold excess of competitor DNA (Figure 5A, lanes 6–9).

Another approach to determine the possible binding preference of H1 for the DNA loop(s) of hcDNA is titration of a mixture of hcDNA and DNA minicircles with increasing amounts of H1. Binding of H1 to hcDNA and small DNA circles was observed at very similar concentrations of histone H1 (notice that the H1-DNA minicircle complexes migrated indistinguishably from that of the linear DNA used for preparation of hcDNA), Figure 5B. hcDNA (in addition to the DNA loop) also contains another possible DNA binding site for histone H1, the DNA hemicatenane (Figure 1D). However, under the conditions of specific and high-affinity binding of HMGB1 to the hemicatenane of hcDNA [28], binding of histone H1 to the DNA hemicatenane was ruled out from DNA footprinting experiments (digestion of the H1-hcDNA complex with exonuclease III, data not shown).

To find out whether the CTD of histone H1 is required for specific binding of the protein to DNA minicircles, EMSA experiments were carried out with peptide H1-CΔ97. Addition of H1-CΔ97 to DNA minicircles resulted in two bands of reduced mobility (Δ1 and Δ2, Figure 5C). We have estimated that the affinity of the H1 peptide for small DNA circles was only ∼2-fold lower (*K_D_* ∼7–9 nM) than that observed with the full-length H1. Specificity of the H1-CΔ97 binding to DNA minicircles was further challenged with unlabeled competitor DNA. While Δ1 was detected up to ∼10^5^-fold mass excess of competitor DNA, the Δ2 band was less stable. Our results indicated that the C-terminal domain was required for specific binding of H1 to the DNA minicircles but it was dispensable for high-affinity binding.

### Modulation of H1 Displacement from DNA by the Redox State of HMGB1

H1 and HMGB1 proteins share not only binding preference to alternative DNA structures, they also occupy similar (if not identical) binding sites within the linker DNA in chromatin (reviewed in [1]). A number of data indicated that linker H1 histones could be excluded or displaced from DNA or chromatin by HMGB-type proteins [14]–[17], suggesting that the presence of both proteins in chromatin might be mutually exclusive. The displacement of linker histones from their binding site could have important biological consequences including local destabilization of chromatin, recruitment of other proteins, and transcriptional activation ([18] and refs. therein). However, mechanism of the HMGB1-mediated displacement of H1 from DNA is unclear.

Here, we have studied whether the HMGB1-mediated displacement of histone H1 from DNA could be modulated by the redox-state of HMGB1. As affinities of H1 or HMGB1 are relatively low for linear DNA [1], we have used in our experiments highly bent and constraint DNA forms (DNA minicircles and hemicatenated DNA loops) for which both proteins exhibit high affinity. In addition, both DNA minicircles and hemicatenated DNA loops possess some structural features imitating the structure of DNA found at the entrance/exit points of the nucleosome.

#### H1-DNA minicircles complex

**Figure 6:**
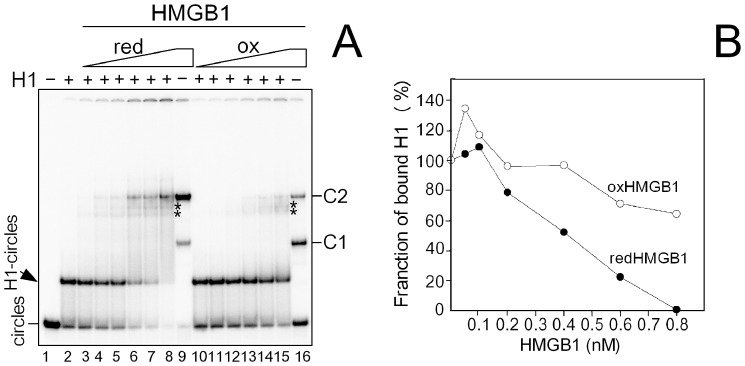
Binding of histone H1 to DNA minicircles is modulated by redox state of HMGB1. **(A**) Titration of the H1-DNA complex with reduced or oxidized HMGB1. ^32^P-labeled 66-bp DNA minicircles (30 pM) were pre-incubated with histone H1 (∼15 nM) and titrated with either reduced or oxidized HMGB1 (0.05, 0.1, 0.2, 0.4, 0.6 and 0.8 nM; lanes 3–8 or 10–15, respectively). HMGB1-DNA complexes prepared at 0.8 nM of reduced (lane 9) or oxidized (lane 16) HMGB1 with no H1 added. Arrowhead denotes the H1-DNA complexes. C1 and C2 denote specific HMGB1-DNA complexes. Asterisks indicate mobility of the ternary H1-HMGB1-DNA complexes. (**B**) Fraction of bound H1 plotted against HMGB1 concentration. Fraction of bound H1 = 100% on axis Y denotes initial amount of bound histone H1 before addition of HMGB1. Concentrations of reduced or oxidized HMGB1 proteins are indicated on axis X. Experiments were performed in triplicates with two different preparations of DNA minicircles and three different amounts of H1. redHMGB1, reduced HMGB1; oxHMGB1, oxidized HMGB1.

Histone H1 was pre-mixed with DNA minicircles of 66-bp, resulting in a discrete band of reduced electrophoretic mobility (Figure 6A, *arrowhead*). Addition of reduced HMGB1 to the H1-DNA complex resulted in gradual disappearance of the complex. We have also observed enhanced formation of the HMGB1-DNA complex as a result of HMGB1 binding to protein-free DNA minicircles. A small fraction of ternary H1-HMGB1-DNA complexes was also detected (Figure 6, asterisks). As the intensity of the ternary H1-HMGB1-DNA complexes was not significantly increased at higher amounts of HMGB1, it is likely that the disappearance of the H1-DNA complex upon addition of HMGB1 was mainly due to H1 displacement rather than due to formation of the ternary complex. Similar experiments performed with oxidized HMGB1 revealed that the protein was significantly less efficient in H1 displacement from DNA than reduced HMGB1 (Figure 6A and B). Higher effectiveness of reduced HMGB1 in H1 displacement from DNA was also confirmed with minicircles of 123-bp, representing much less distorted DNA as compares to 66-bp minicicles (not shown).

#### H1-hcDNA complex

**Figure 7:**
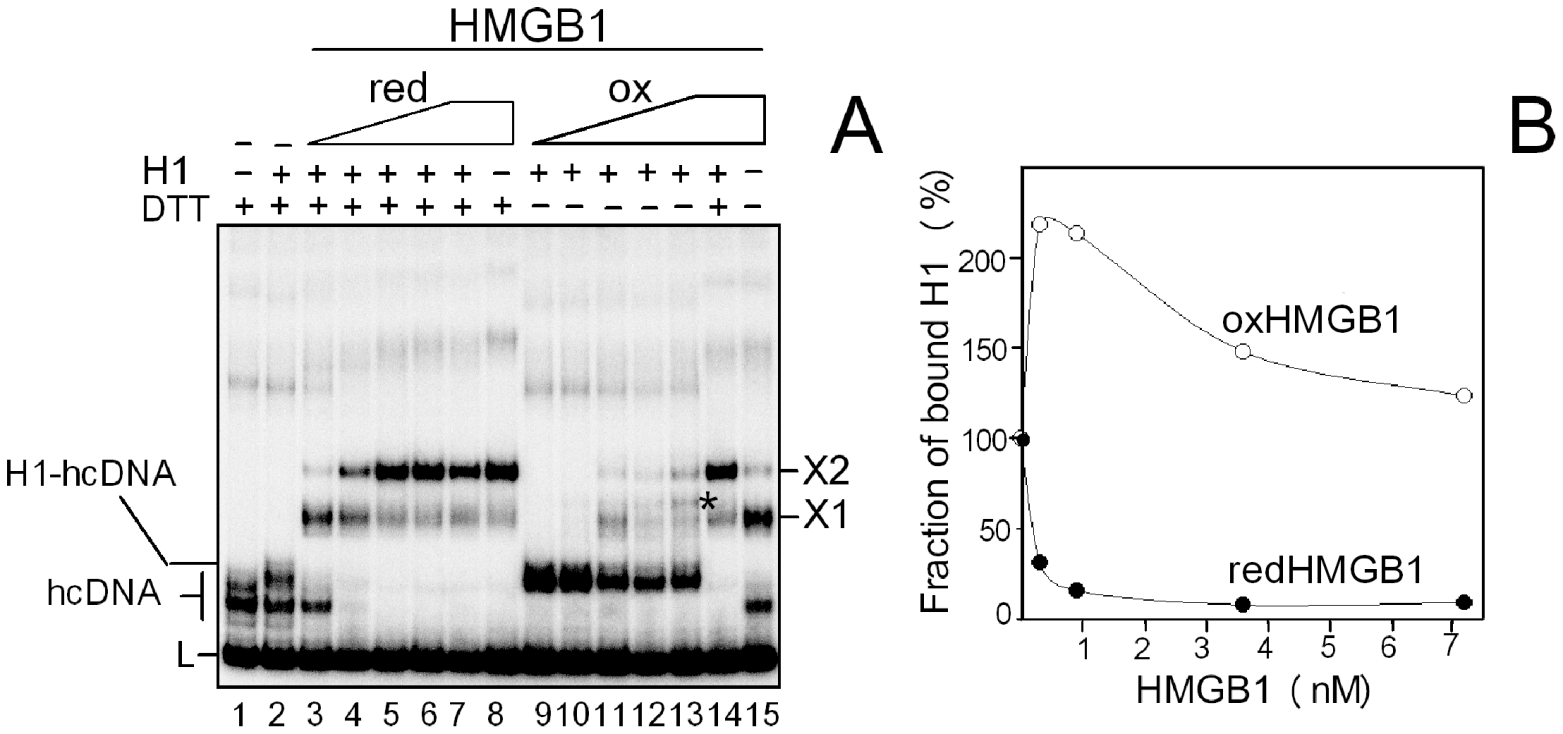
Binding of histone H1 to hcDNA is modulated by redox state of HMGB1. (**A**) Titration of the H1-hcDNA complex with reduced or oxidized HMGB1. ^32^P-labeled hcDNA (15 pM) was pre-incubated with histone H1 (∼1 nM, lane 2) and titrated with either reduced or oxidized HMGB1 (0.3, 0.9, 1.8, 3.6 and 7.2 nM; lanes 3–7 and 9–13, respectively). Lane 8, redHMGB1-hcDNA complex (prepared at 7.2 nM HMGB1) with no H1 added. Lane 15, oxHMGB1-hcDNA complex (prepared at 7.2 nM HMGB1) with no H1 added. Lane 14, oxHMGB1-H1-hcDNA complex from lane 13 treated with 10 mM DTT. L, linear fragment used for the preparation of hcDNA. Asterisk indicates electrophoretic mobility of the ternary complex H1-HMGB1-hcDNA. X1 and X2 denote the specific HMGB1-hcDNA complexes. (**B**) Fraction of H1 bound to hcDNA plotted against HMGB1 concentration. Fraction of bound H1 = 100% on axis Y denotes initial amount histone H1 bound to hcDNA before addition of HMGB1. Concentrations of reduced or oxidized HMGB1 proteins are indicated on axis X. Experiments were performed in quadruplicates with two different preparations of hcDNA. redHMGB1, reduced HMGB1; oxHMGB1, oxidized HMGB1.

To find out whether the ability of HMGB1 to displace H1 from DNA minicirles could be also observed with H1 bound to other highly bent DNA, similar experiments were performed with complexes of H1 bound to hemicatenated DNA loops (hcDNA). As shown in Figure 7A, striking differences were observed upon titration of the H1-hcDNA complex with reduced or oxidized HMGB1. While reduced HMGB1 could easily displace H1 from binding to hcDNA (lanes 3–7), no displacement of H1 was observed upon addition of oxidized HMGB1 to the H1-hcDNA complex (lanes 9–13). Interestingly, low amounts of oxidized HMGB1 could, on the other hand, promote histone H1 binding to DNA (Figure 7B). We have also demonstrated that addition of DTT to the H1-oxHMGB1-hcDNA complex resulted in complete displacement of H1 from DNA (Figure 7, lanes 13 and 14, and also 7), providing further evidence that the binding properties of HMGB1 are reversibly modulated by redox state of HMGB1.

While no ternary H1-HMGB1-hcDNA complex was detected with reduced HMGB1, only a faint band corresponding to the ternary complex was seen upon addition of higher amounts of oxidized HMGB1 to the H1-hcDNA complex (Figure 7A, asterisk). The ternary H1-HMGB1-DNA complex (observed both in Figures 6 and 7) may be in principle a consequence of direct protein-protein interactions of HMGB1 with the DNA-bound histone H1 (interaction of HMGB1 with linker histones in free solution or linker histones bound to DNA has been reported previously, refs. [15], [18], [32]). It can also arise from HMGB1 binding to other binding sites on small DNA circles or hcDNA. For example, hcDNA contains, in addition to the DNA loop, the DNA hemicatenane for which HMGB1 exhibits an extreme affinity ([28] and refs. therein). Thus, binding of HMGB1 to the hemicatenane can occur without displacing histone H1, assuming H1 binding to the DNA loop of hcDNA.

### Interaction of HMGB1 with Histone H1

**Figure 8:**
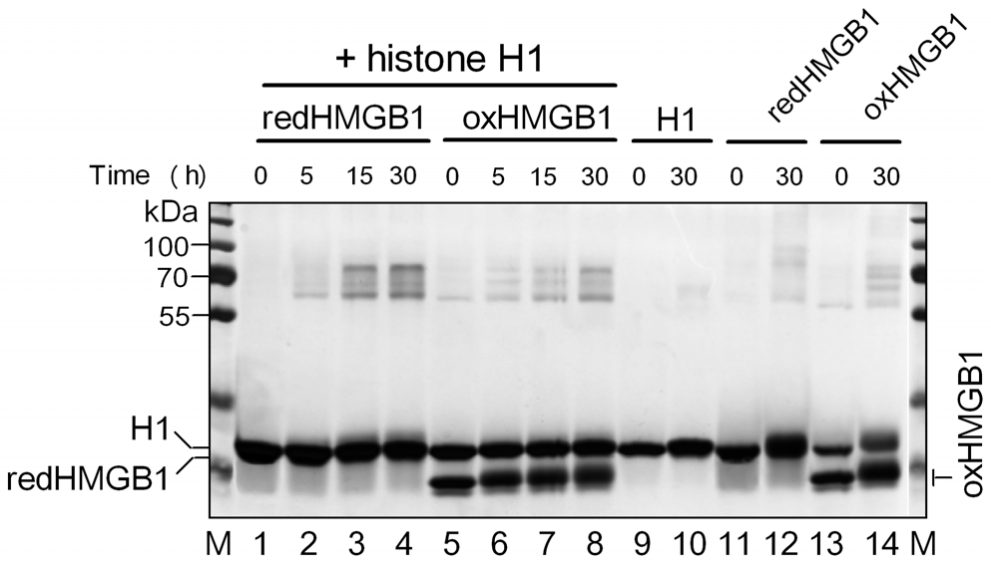
Binding of reduced and oxidized HMGB1 to histone H1 in free solution. H1 was mixed with reduced HMGB1 (lanes 1–4) or oxidized HMGB1 (lanes 5–8) at a molar ratio 1:1 and treated with dimethyl suberimidate for the times indicated. Cross-linking of (individual proteins) H1 (lanes 9–10), redHMGB1 (lanes 11–12) and oxHMGB1 (lanes 13–14) is shown. M, molecular mass marker; redHMGB1, reduced HMGB1 protein; oxHMGB1, oxidized HMGB1 protein. Gel was stained with silver.

Previous report demonstrated the ability of linker histones H1 to interact with HMGB1 free in solution [15], [18], [32]. Here, we have tested whether the limited ability of the oxidized HMGB1 protein to displace H1 from DNA (Figures 6 and 7) may be related to altered HMGB1-H1 interactions. We have used dimethyl suberimidate cross-linking to study the impact of mild oxidation of HMGB1 on the protein binding to histone H1. At a 1:1 molar ratio, H1 was cross-linked to HMGB1 to produce heterodimers and heterotrimers as the main products in a time-dependent manner (Figure 8). Although individual proteins could produce some cross-linked products (homopolymers), their mobility was mostly distinct from the H1-HMGB1 heterodimers/heterotrimers and their formation diminished when H1 was present in the course of cross-linking. The main conclusion from the cross-linking experiments shown in Figure 8 is that the intensity of the H1-oxHMGB1 heterodimers/trimers was visibly reduced relative to similar cross-links with reduced HMGB1, indicating lower tendency of oxidized HMGB1 to bind H1 in free solution (chemical cross-linking can not, however, provide any details regarding the nature of the H1-HMGB1 interactions). Thus, the observed lower affinity of oxidized HMGB1 for H1 could be one of the factors contributing to limited ability of oxidized HMGB1 to displace H1 from DNA (*see* the Discussion).

## Discussion

In this paper we report a high affinity binding of histone H1 to small DNA circles and hemicatenated DNA loops. We have also demonstrated that the ability of HMGB1 to displace histone H1 from DNA is modulated by the redox-state of HMGB1. While reduced HMGB1 could easily displace H1 from DNA, oxidized HMGB1 had limited capability to displace H1.

### Biological Relevance of Histone H1 and HMGB1 Binding to DNA Crossings

The high affinity of HMGB1 and H1 to DNA minicircles or hemicatenated DNA loops (hcDNA) presumably reflects a high degree of complementarities between the DNA-binding faces of the proteins and the latter DNA structures ([28], reviewed in [1]). It is intriguing that the two major chromatin architectural proteins, H1 and HMGB1, bind to DNA structures such as synthetic four-way junctions (4WJs) [33], [34], hemicatenanes [26]–[28], and bent (distorted) DNA such as DNA modified by anticancer drug cisplatin ([29], [35] or small DNA circles (this paper and [24]. Thus, HMGB1 and histone H1 share some degree of capacity to bind to DNA crossings and bent DNA, to bend and unwind DNA upon binding (reviewed in [1], [36]). The binding similarities of H1 and HMGB1 are unexpected due to the presence of unrelated DNA binding motifs and completely different structures of the proteins.

There are certainly more H1 and HMGB1 molecules in eukaryotic cell (∼1 H1 molecule per nucleosome and 1 molecule of HMGB1 for 10–15 nucleosomes, [1]) than hemicatenanes, Holliday junctions (4WJs) or other alternative DNA structures, making them unlikely primary binding sites for these proteins. However, it is possible that some of these DNA structures may mimic their (not yet identified) *in vivo* DNA binding sites due to high complementarities of their binding surfaces with the DNA-binding regions of the proteins. DNA crossings may imitate the structure of DNA found at the entrance/exit points of the nucleosome.

### Competition of H1 and HMGB1 for Binding Sites on DNA and Chromatin

Early experiments revealed that vertebrate HMGB1 interacts *in vitro* with linker histones, including histone H1 (refs. in [18] and [1]). Consequences of HMGB1-H1 interactions were demonstrated on minichromosomes in murine cells over-expressing HMGB1, revealing enhanced susceptibility to micrococcal nuclease digestion and reduced amounts of histone H1 [37]. Soluble chromatin, released from mouse myeloma nuclei upon micrococcal nuclease digestion, contained mononucleosomes depleted in histone H1 but highly enriched in HMGB1 [14]. Substitution of HMGB-type proteins by histone H1 was also reported in early embryogenesis (refs. in [18]). In addition, weakening of H1 binding to chromatin or even its displacement was demonstrated from microinjection of HMGB1 into living cells expressing histone H1 fused with GFP [38]. The above data suggested mutually exclusive binding of H1 and HMGB1 in the nucleosome (refs. in [1]).

Histone H1 exhibits significantly higher affinity for reconstituted dinucleosomes than HMGB1 (*K*_d_ of 7.4 and 300 nM, respectively) [39]. On the other hand, H1 exhibits lower affinity for hcDNA or DNA minicircles than HMGB1 (this report), enabling replacement of H1 by HMGB1. It is unclear whether only DNA binding affinities of HMGB1 and histone H1 are decisive in mutual competition of the proteins for DNA binding or whether other factors such as direct interaction of HMGB1 with H1 and/or core histones in nucleosomes could contribute to H1/HMGB1 competition for their binding sites in chromatin (refs. in [1], [18]). Accessibility of the basic C-terminal terminus (CTD) of H1 in the complex with DNA may modulate direct physical interaction of H1 with the acidic C-terminus of HMGB1 [18]. The accessibility of the acidic C-tail of HMGB1 for H1 binding can also be an important factor in modulation of the HMGB1-H1 interactions (as the structures of redHMGB1 or oxHMGB1 have not yet been published, we can only speculate whether the oxidization-mediated conformational change of HMGB1 could result in lower availability of the acidic C-tail of the protein [18] for the interaction with histone H1). Thus, binding of H1 with HMGB1 can enhance the affinity of HMGB1 for DNA and, subsequently, lower the affinity of H1 for DNA. Finally, affinity of H1 and HMGB1 for DNA/chromatin can be fine-tuned by post-translation modifications of the proteins (reviewed in [1], [40]), as well as by the redox-sensitive cysteine residues of HMGB1 (this paper).

In conclusion, we have demonstrated that redox state of HMGB1 can significantly modulate the ability of the protein to bind and bend DNA, as well as to interact with histone H1. We have also shown that reduced HMGB1 can efficiently displace histone H1 from DNA, while oxidized HMGB1 has limited capacity for H1 displacement. Our experiments suggest a novel mechanism for reversible modulation of H1 binding to DNA by the redox state of HMGB1. Conformational change of HMGB1 upon oxidization (due to formation of the disuphide bridge between Cys 23 and 45 within the domain A, Figure 1B) could constitute an important mechanism contributing to competition between HMGB1 and H1 for their binding sites in chromatin [2]). Replacement of linker histones H1 from their binding sites in chromatin (recently demonstrated on a single nucleosome [41]) might have important biological consequences, including local destabilization of chromatin, recruitment of other proteins, and transcriptional activation.

## Materials and Methods

### Plasmids and Competitor DNA

Competitor DNA was either sonicated double-stranded salmon sperm DNA (ds-DNA), λ-DNA or a 75bp duplex derived from the intronic IgH enhancer (MARs). The latter DNA duplex was prepared by slow annealing of the following oligonucleotides: Top, 5′-TCTTTAATTTCTAATATATTTAGAATCTTTAATTTCTAATATATTTAGAATCTTTAATTTCTAATATATTTAGAA-3′; Low, 5′-TTCTA-AATATATTAGAAATTAAAGATTCT-AAATATATTAGAAATTAAAGATTCTAAATATATTAGAAATTAAAGA-3′.

### Preparation of hcDNA and DNA Minicircles

Hemicatenated DNA (hcDNA) was prepared from an 120-bp *ClaI*-*EcoRI* restriction fragment containing a 60-bp tract of poly(CA).poly(TG), labeled at 5′-termini by [γ-^32^P]-ATP as detailed in [27]. hcDNA was kindly provided by François Strauss (National Museum of Natural History, Paris, France). Labeled hcDNA was kept at +4°C for up to 1 month. DNA minicircles of 66- or 123-bp were prepared as in [19], [27], with the exception that HMGB1 (A+B) di-domain was used to promote ligase-mediated circularization of the 66-bp linear DNA duplex.

### Antibodies

Mouse monoclonal anti-histone H1^o^ (sc-377468X) and rabbit polyclonal anti-HMGB1 (ab18256) antibodies were purchased from Santa Cruz or Abcam, respectively.

### HMGB1

His-tagged recombinant wt HMGB1 or HMGB1(A+B) di-domain were expressed in *E. coli* BL21(DE3) cells from plasmids encoding rat cDNAs (the amino acid sequence of the expressed rat HMGB1 is identical to that of the human protein) ([19]). Calf thymus HMGB1 was isolated under non-denaturing conditions as detailed in [15]. Untagged wt HMGB1 and HMGB1(C106S) mutant were expressed from pETite-N-his-SUMO-rHMGB1 constructs prepared by using “Expresso T7 SUMO Cloning and Expression System” (Lucigen) and “Site-directed Mutagenesis Kit” (ThermoFisher). Identity of HMGB1 sequences in all plasmid constructs was verified by dideoxy-sequencing on both strands. HMGB1 was oxidized under mild conditions to promote disulfide bond formation as detailed in [5], [9]. Briefly, HMGB1 was dialyzed overnight against DB buffer (0.15 M NaCl, 20 mM Tris. HCl pH 8.0, 1 mM PMSF) containing 5 µM CuCl_2_, followed by re-dialysis against DB buffer lacking CuCl_2_. In some experiments, the oxidized HMGB1 protein was reduced by treatment with 10 mM DTT at 30°C for 30 min. The concentrations of the purified proteins (all proteins were pre-treated with 10 mM DTT) were determined from the SDS-PAGE gel by the Coomassie Brilliant Blue G-250 Protein Assay (Bio-Rad) using BSA as standard and/or mass-spectrometry.

### MALDI-TOF Mass-Spectrometry

Identity of HMGB1 (24.89 kDa, 215 amino acids including the N-terminal methionine), as well as formation of disulphide bridge between cysteine residues 23 and 45, was determined by MALDI-TOF mass-spectrometry. Briefly, HMGB1 proteins were subjected to trypsin digestion. The digests (1 µl) were mixed with CHCA (α-cyano-4-hydroxycinnamic acid) matrix solution on the Anchor Chip target in a 2:1 ratio. The digests were analyzed by using an Ultraflex III MALDI-TOF mass spectrometer (Bruker Daltonik, Bremen, Germany). An external calibration procedure was employed, using a mixture of seven peptide standards (Bruker Daltonik). Peptide maps were acquired in reflectron positive mode (25 kV acceleration voltage) with 800 laser shots and peaks with 0.70–4.1 kDa mass range and minimum S/N 10 were picked out for MS/MS analysis employing LID-LIFT arrangement with 600 laser shots for each peptide. The Flex Analysis 3.4 and MS Biotools 3.2 (Bruker Daltonik) software were used for data processing. MASCOT 2.4 (MatrixScience, London, UK) search engine was used for processing the MS and MS/MS data. Database searches were done against the NCBI protein database. A mass tolerance of 100 ppm was allowed during processing MALDI MS data for PMF and 0.6 Da during processing LID-LIFT data for MS/MS ion searches. The MS/MS data of peptides containing disulphide bridges were interpreted manually.

### Histone H1 and Truncated Forms

Recombinant human histone H1^o^ (referred throughout the manuscript as histone H1) was purchased from New Egland BioLabs. Mouse histone H1^o^ and the CTD-truncated mutant were expressed from plasmids pET-H1^o^-11d and pET-H1^o^-CΔ97-11d, respectively (the plasmids were kindly provided by Jeffrey C. Hansen, Colorado State University, Fort Collins, USA). Recombinant mouse histone H1^o^ and its truncated form H1-CΔ97 (corresponding to histone H1^o^ lacking the C-terminal amino acid residues 97–193) were expressed in *E. coli* BL21(DE3) cells and purified on the CM-Sephadex C-25 column as detailed in [31]. The obtained fractions were analyzed by SDS/15%-PAGE, and the fractions containing purified proteins were combined and dialyzed against dialysis buffer (10 mM Tris pH 8.0, 1 mM EDTA and 50 mM NaCl). The concentrations of the purified proteins were determined from the SDS-PAGE gel by the Coomassie Brilliant Blue G-250 Protein Assay (Bio-Rad) using BSA as standard.

### The Ligase-mediated Circularization and DNA End-joining Assays

The assays were performed as previously described [19], [27]. Briefly, the ^32^P-labeled 66-bp (*Nde*I ends) or 123-bp (*Ava*I ends) DNA duplexes (∼0.2 nM or 2 nM for circularization or DNA end-joining assays, respectively) were ligated by T4 DNA ligase (0.05 units, Takara) in the absence or presence of HMGB1 (typically 6–300 nM) at 30°C for 40 min. Termination of ligation and treatment of samples with Proteinase K was performed as previously reported [19]. Some of the samples were digested (before Proteinase K treatment) with 4–20 units of exonuclease III (Promega) at 37°C for 30 min. Protein-free DNA samples were resolved on pre-run 5% polyacrylamide gels in 0.5xTBE buffer (250 V for 4 hs at 4°C) and DNA was visualized and quantified from dried gels on PhosphorImager Typhoon SLA9000 (GE).

### Protein-DNA Binding Studies

Electrophoresis Mobility Shift Assay (EMSA) was carried out with ^32^P-labeled DNA in a total volume of 25 µl in 1× EMSA buffer containing 50 mM NaCl, 25 mM Tris-HCl, pH 7.5, 1 mM EDTA, 1 mM DTT, 100 µg/ml acetylated BSA, and 3% glycerol. Reaction mixtures containing ^32^P-labeled DNA (with or without unlabeled competitor DNA) and proteins were pre-incubated on ice (unless otherwise indicated) for 30 min. Super-shift experiments were carried out by addition of 0.5–1 µL of specific antibody to the pre-incubated protein-DNA complexes, followed by 20 min incubation on ice (in the presence of a 1000-fold mass excess of unlabeled competitor DNA over ^32^P-labeled DNA). Reaction mixtures were finally loaded on pre-run 5% or 8% polyacrylamide gels (29:1 acrylamide/N,N′-methylenebisacrylamide) in 0.5x TBE containing at 200 V (4°C) for 4–6 h. Following the electrophoresis, the gels were dried, and the DNA visualized and quantified on PhosphorImager Typhoon SLA9000 (GE). The dissociation constant *K*_d_ was approximated as the total protein concentration at the point of the titration where the fraction of the protein-bound DNA was 0.5 [9], [19].

### Chemical Cross-linking

Dimethyl suberimidate (DMS; Pierce) was dissolved at 10 mg/ml in 10 mM sodium phosphate pH 7.5, and 1/10 volume was added to histone H1, HMGB1 or 1:1 H1/HMGB1 mixtures (final concentration of proteins ∼1 µM) in 0.05–0.15 M NaCl containing 10 mM sodium phosphate pH 7.5 (1 mM DTT was included for cross-linking of reduced HMGB1). Samples (20 µl) were cross-linked with DMS at 24°C for different times (as indicated) and reactions were terminated by addition of a mixture containing 2 µl of 1M glycine and 3 µl of 10×SDS loading buffer. Proteins were then immediately heated in a water-bath, loaded on to SDS/15%-polyacrylamide gels, and the gels stained with silver.
